# Implications of assisted dying for nursing practice

**DOI:** 10.1177/09697330251314096

**Published:** 2025-01-16

**Authors:** Mark Wareing

**Affiliations:** 3890 Brunel University of London

**Keywords:** Assisted dying, assisted suicide, nursing practice

## Abstract

This conceptual paper considers the practice implications of assisted dying for contemporary nursing practice within the United Kingdom in response to the publication of a parliamentary report leading to a private members’ bill that will form the basis of a debate and possible change in legislation. A recurring theme within the nursing research is how nurses should respond to patients expressing an interest or making a request for assisted dying. This paper explores contemporary evidence and argues that the procedure of assisted dying is a complex (manifold) and puzzling (paradoxical) practice. The UK nursing profession may replicate recent healthcare catastrophes if the response to a proposal for assisted dying is based on a technical-rational stance, or if nurses merely coalesce around a single determinant such as patient autonomy. The paper presents two nursing communicative interventions that seek to address how to respond to a patient request for an assisted death that foregrounds the preferences and personhood of the patient whilst providing opportunities for enquiry-based approaches to enhance nursing responses to intractable suffering.

## Introduction

In March 2024, the United Kingdom (UK) parliament’s House of Commons Health & Social Care committee published its inquiry report into assisted dying.^1^ One of the nine terms of reference was to consider the professional and ethical considerations involved in allowing physicians to assist someone to end their life. The inquiry report received verbal as well as written evidence from a wide range of healthcare professionals, academics, politicians and members of the general public and generated recommendations to serve as a basis for discussion and debate in future parliaments. At the time of writing the Terminally Ill Adults (End of Life) Bill for England and Wales is proceeding to the committee stage, following a 2^nd^ reading (debate) and the outcome of a free vote by members of parliament.^2^

This paper will consider the implications of the introduction of assisted dying for contemporary clinical nursing practice, including the ethical, practice and communicative implications of patients making a request to nurses for assistance in dying.

## Assisted dying

Assisted dying and medical assisted in dying (MAiD) have been legalised in an increasing number of countries including Australia (the state of Victoria); the United States of America (Washington, Montana, Oregon, Vermont, California, Colorado and Hawaii); Europe (Netherlands, Belgium, Luxembourg, Spain and Germany) and Columbia.^3–5^ Canada introduced legislation in 2016 which permitted nurse as well as medical practitioners to act as assessors and providers of MAiD and has been the focus of illuminating research into the complexities of implementing the procedure.^6–9^ At the time of writing legislation is being considered in Scotland and the Crown Dependencies of Jersey, Guernsey and the Isle of Man; which although geographically close to the United Kingdom have autonomous governing bodies and separate legislative frameworks.

The geographical implications of the provision of assisted dying are relevant to clinicians working in rural, remote areas where access to healthcare maybe limited and lone working is common.^10^ It could be argued that legislation introduced in Canada including a distinct role for nurse practitioners as assessors and providers of assisted dying,^11^ was influenced by the need for disparate populations to gain access to healthcare.^12^ Interestingly, current assisted dying legislation being considered in Scotland^13^ appears to mirror Canadian law, with a role for registered medical practitioners and registered nurses (referred to as ‘authorised health professionals’) who also serve communities in rural areas such as the islands and highlands.

Nurses who support and engage in assisted dying are likely to have less years of clinical experience, work predominantly in acute care, have a higher level of education^14,15^ or are nurses who happen to be male.^16^ Nurses who have lower levels of internalised religiosity or spirituality are less likely to object to assisted dying although nursing staff with strong ethical and philosophical stances may support assisted dying.^17–19^ Whilst the participation of nurse practitioners as assessors and providers of assisted dying is legal in Canada, research features accounts from nurses who have disclosed active participation in euthanasia within countries where it is not legal for them to do so.^19,14^

### Nursing roles

The roles of Healthcare Assistant (HCA), Nursing Associate (NA) and Registered Nurse (RN) are well established within the UK and represent levels of practice from the unqualified skilled helper^20^ to registered paraprofessional and the registered nurse with protected title status.^21^ The care of the dying is a key nursing responsibility as reflected in the UK Nursing & Midwifery Council (NMC) tandards of proficiency for registered nursing associates^22^ which require:

‘…an understanding of how to deliver sensitive and compassionate end of life care to support people to plan for their end of life, giving information and support to people who are dying, their families and the bereaved’ (Standard 3.13, 22 pg. 16)

Nursing associates are also required to:

‘review preferences and care priorities of the dying person and their family and carers, and ensure changes are communicated as appropriate’ (Standard 9.2, 22 pg. 33).

The standards of proficiency^23^ require registered nurses to:

‘demonstrate the knowledge and skills required to prioritise what is important to people and their families when providing evidence-based person-centred nursing care at end of life including the care of people who are dying, families, the deceased and the bereaved’ (Standard 4.9, 23 pg. 21). Additionally, registered nurses must:

‘assess and review preferences and care priorities of the dying person and their family and carers’ (Standard 10.3, 23 pg.43).

The NMC standards emphasise care that is characterised not only by person-centredness but the priorities of patients, their families and carers, whilst delivering interventions that are evidence-based. Regardless of the structure of the nursing hierarchy, each distinct role generates a particular lived experience for the clinician which has been described as a way of being that operates within a particular lifeworld^24^; the lifeworld of clinical nursing practice. This phenomenological approach which recognises the lived experience of practice, suggests that engagement in clinical care is profoundly meaningful and constitutes the role of the HCA, NA and RN; not only by what they do, but who they are.^25–27^

### Nursing as practice

A particular feature of contemporary clinical nursing practice within the United Kingdom is the division of labour which persists between qualified and unqualified nursing staff^28^; in particular the relationship between the scope of practice of healthcare assistants who primarily deliver direct patient care and registered nursing staff whose workload disproportionally features the management of medication and administrative activity.^27^ One observation is that clinical ‘face workers’ defined as the human face of the helping services ‘…the faces that are known…those who work directly with users that have something in common: the face-work relationship’^29^ (pg.7–8), tend to be some of the least qualified members of the nursing team. The role of support workers such as healthcare assistants are characterised by a range of tasks that were traditionally categorised as ‘dirty and emotional work’ which includes dealing with death as a routine part of working life in situations that are highly contingent, situational and deeply meaningful.^20^

This factor has considerable implications for assisted dying as a key theme arising from research relates to patients making requests to end their life to nurses.^30–32^ The practice implications are that nursing staff are required to respond in a professional, sensitive, compassionate and caring manner in accordance with their professional codes of conduct, although in some countries where assisted dying is legal, registered nurses have to exercise care not to introduce euthanasia as an option to patients, in order to meet the requirements of legislation.^31^

### Ethical practice

Nursing interventions are delivered within a wide landscape of health and social care practice influenced by historical, societal and pedagogical features. Hannah Arendt argued that work should be differentiated from mere labour, as work authenticates the human condition,^33^ but warned that technocratic bureaucracy^34^ can lead to ‘non-thinking’. For Arendt, thinking was not just a way of being a person it was a way of having an internal dialogue which formed the contemplative life that should accompany the active working life, in contrast to the functionary, unquestioning job holder. A technical-rational response to a request from a patient for assisted dying where nurses merely adopt a stance of responding to any expressed need from the patient has been criticised as rendering the role of the nurse as a death administrator^35^ and threatens to recast the nursing profession as a mere service industry. Respect for autonomy is characterised by an individual’s right to self-determination including the right of a competent person to refuse medical treatment.^36^ Whilst assisted dying is regarded as providing an autonomous decision for patients to end their life at a time and manner of their choosing, this view frames autonomy in an individualistic and subjective manner. Keown^37^ argues that once autonomy is reinterpreted as respect for individual choice, other standards such as the sanctity and inviolability of life may be demoted or discarded since they are not chosen by individuals.

Arendt’s work has attracted a resurgence of interest in the context of a series of healthcare catastrophes within the UK.^38,39^ Her concept of ‘non-thinking’^40^ is relevant to a particularly challenging recent healthcare catastrophe where clinicians and therapists were heavily criticised for failing to safeguard children and young people who were questioning their gender identity or experiencing gender dysphoria at the now closed Gender Identity Development Service (GIDS) based at the Portman and Tavistock NHS Foundation Trust in London. The Cass Review^41^ found that staff adopted an unquestioning affirmative approach towards children and young people unsure of their gender which over shadowed other issues such as mental health, abuse and other complex trauma and had departed from an earlier service commitment towards audit and evidence-based practice.^42^ The review suggested that clinicians were operating within an environment that was heavily influenced by political and ideological concerns. In common with other contemporary healthcare catastrophes associated with moral blindness,^43^ despite concerns raised by several healthcare professionals employed by GIDS, the prescription of ‘puberty-blockers’ became the predominant model of treatment of young people with gender dysphoria. An additional factor was direct pressure that GIDS staff experienced from a parental support group (Mermaids), which created an ideological basis for treatment and management decisions.^44^

### Assistance in dying

In countries where assisted dying has been legalised, clinical staff report an absence of clear operational policies which renders the practice problematic when there is uncertainty around the interpretation of legislation.^45^ Nurses are concerned that their participation in assisted dying may infringe guidance from professional standard regulatory bodies; particularly in the area of responding to patient requests.^46–48^ Whilst nurses recognise that standard operating practices afford safeguarding protection for patients, administration of the documentation can make the provision of assisted dying highly time dependent in the context of the window permitted for the completion of the procedure and the clinical condition of the patient; in particular, a patient’s ability to give final verbal consent and to self-administer medication.^48^

Qualitative research findings provide insight into the need for clinical nursing staff who have participated in the procedure to receive professional as well as psychological debriefing in order to process the highly emotive and profound nature of the procedure including the care delivered to the family.^7,8,10^ Some nurses who have participated or witnessed assisted dying have described their experience as positive^6^, although research suggests that engagement can cause moral distress, deep and strong emotional responses with some nurses fearing that their involvement may lead to desensitisation.^48^

In order to consider the implications for the nursing family, two conceptual frameworks are proposed in order to make sense of the practice implications of participation in assisted dying. The practice will be conceptualised as ‘manifold’, from a macro perspective, considering the range of components leading to the death of a patient; and a ‘paradoxical practice’ from the perspectives arising from the voices of nurses (the micro perspective) as suggested by contemporary research findings.

### Manifold practice

The word manifold describes an object that is varied or diverse in appearance, comprising of component parts and relations that perform several functions at once in a complex or difficult manner.^49^ An example would be an exhaust manifold within an internal combustion engine which collects exhaust fumes released from the engine cylinders and directs them to the catalytic converter.

Assisted dying is a manifold practice as it comprises of deep complex moral, ethical, philosophical components that coalesce around a single irretrievable, final event that draws on a wide range of moral, social, emotional and psychological resources amongst members of the nursing family. The practice is unidirectional, singular, profound and highly controversial in the context of ethical, philosophical, religious and spiritual beliefs and convictions; both for the conscientious participator^55^ and conscientious objector.^50^

### Paradoxical practice

Hébert & Asri^51^ describe the involvement of nurses in assisted dying as a paradoxical practice in terms of the lived experience of clinical nursing staff, as typified by the sudden nature of the planned/scheduled death and the surprising and somewhat unexpected emotional experiences of nursing staff including issues of doubt and existential concern.^48^ Paradoxically, in the context of the procedure, research into the role of nursing staff in the management of refractory symptoms and suffering^52^ seems to be largely absent despite evidence suggesting that the fear of suffering and unacceptable symptoms trigger patient requests for an assisted death.^53^

Assisted dying is contingent upon a medical practitioner’s prescription of a lethal dose of a sedative, analgesic, cardiotoxic agent and/or antiemetic^54^ which requires the participation of nurses in the preparation, management and supervisory administration (or in Canada) direct administration of medication for the sole purpose of termination of life via an intravenous infusion or oral administration. Given that the median time from administration of intravenous (IV) medication to death can be up to 9 minutes,^55^ it is not clear from the literature what the role of a registered nurse would be if an intravenous infusion stopped or an IV pump failed, due to occlusion or poor peripheral vascular access.

Effective learning within workplace and clinical environments is contingent upon a participatory learning approach for nursing students or indeed any member of the nursing family in order to become a safe, proficient and competent practitioner.^56^ Participatory learning^57^ requires the learner to have gradual exposure to the learning affordance or activity with the support of a practice supervisor who may adopt a scaffolded learning approach^58^ to ensure that the learner can safely and supportively engage in a clinical procedure and demonstrate their competence prior to going solo. Participatory learning becomes deeply problematic for any member of the nursing family; particularly those who are least experienced and junior if the learner is unsure of their stance on assisted dying^59^ and encouraged to observe or even partake in the procedure by virtue of needing experience in intravenous medication management and the care of the dying.^22,23^

The provision of protection for conscientious objectors; nurses who do not wish to take part in assisted dying, is recognised in international and proposed UK legislation,^2^ although the operational nature is problematic as nurses report varying degrees of support from employers including being offered a day-off to only being permitted to leave the room immediately prior to a patient receiving the procedure.^60,61^ The experience of nurses as conscientious objectors is deeply conflicted as nursing staff feel that they may be withdrawing support for their clinical team, fear accusations of abandoning their patients in addition to being asked to provide a rationale for their objection, which may require the disclosure of an orthodox religious conviction contrary to prevailing secular liberal or progressive world views.^62^

Assisted dying is said to be a paradoxical practice, not least because the literature points to a research priority in the area of nursing intervention in the management of refractory symptoms and refractory suffering. Research during end of life care could be undertaken in a non-invasive manner using methodologies such as documentary and observational research^63,64^ that uncover the nature of clinical nursing practice, diagnostic reasoning, decision-making, therapeutic intervention, person and family centred care.

It is paradoxical that a recurrent theme within the literature is the need to respond to patient requests for assisted dying rather than conversations with patients as well as their family and friends when dying and the episode of death is anticipated. What is also paradoxical is evidence from the literature that suggests that relatively small numbers of patients who express an interest in assisted dying undertake the procedure.^65,66^ This may be due to patient deterioration, the administrative frameworks necessary to safeguard the wellbeing of the patient or the initiation of end of life care sufficient for assisted dying to be no longer necessary.^67^

As highlighted earlier, a failure to appreciate the limits of autonomy may diminish equally valid ethical principles such as beneficence, the norm that promotes beneficial action whilst balancing risks with costs.^36^ The United Nations Universal Declaration of Human Rights^68^ affirms dignity rather than a right to autonomy and serves as a reminder that the prohibition of an autonomous choice, even with a person’s free consent, is sometimes necessary to protect young and vulnerable people as reflected in the conclusions of the Cass Review.^41^

In the light of both the manifold and paradoxical nature of assisted dying, two clinical nursing interventions are considered that centre on communicative actions to initiate conversations around death and dying with patients approaching end of life care and a philosophical approach which recognises the fluid and dynamic nature of a patient’s personhood and desires^71^ as they approach the end of life.

Communicative actions within end of life care are known to be characterised by the need for patient’s perceptions to be re-evaluated overtime as choices may fluctuate in response to mood, self-identity and engagement with care givers.^65^ Both interventions are based on a positive, realist, person-centred stance that seeks to balance patient autonomy with considerations relating to the relational interdependence of the patient; their care situated within a nursing family, whilst retaining opportunities for practice enquiry during end of life care to alleviate refractory symptoms and suffering ahead of a peaceful death.

### Communicative intervention

Repositioning autonomy and dignity to create end of life care that is beneficent would seem particularly important when shared decision making is required, particularly when a patient has cited their frailty as the basis for a request for an assisted death and families wish to engage in pre-emptive planning to avoid a hospital admission.

The ‘criteria for screening and triaging to appropriate alternative care’ (criSTAL) tool^69^ has been used by nursing and medical staff to undertake a comprehensive assessment of patients in order to identify end of life status prior to hospitalisation (see Table 1). The tool features eight questions relating to the biomedical status of the patient and criterion relating to medical history that are regarded as a risk factor or predictor of short to medium term death including evidence of frailty and the social circumstances of the patient. The aim of the tool is to provide an objective assessment of the dying patient as a starting point for an honest conversation with patients and their families and recognises that dying is part of the life cycle. A key rationale for the development of the tool was to avoid moral fanaticism^70^ characterised by potentially harmful and futile clinical intervention. In a prospective study of 249 patients with a median age of 78–80 years, the tool was found to have good discrimant ability when used in Australia and Denmark by providing a ‘red flag’ for high risk and the probability of death based on risk factors.^71^ The authors concluded that criSTAL scores were not intended to dictate management but initiate a conversation that might transition a patient from active to palliative or comfort care, where appropriate, at a threshold where the harm/benefit ratio of having end-of-life care was optimal.

**Table 1.** Components of the criSTAL tool (Cardona-Morrell & Hillman,^73^ pg. 85, 2015).

• Age 65 or over
• Being admitted via emergency this hospitalisation (associated with 25% mortality within 1 year) OR Meets 2 or more of the following deterioration criteria on admission
1. Decreased LOC: Glasgow coma score change >2 or AVPU = *p* or U
2. Systolic blood pressure <90 mm Hg
3. Respiratory rate <5 or >30
4. Pulse rate <40 or >140
5. Need for oxygen therapy or known oxygen saturation <90%
6. Hypoglycaemia
7. Repeat or prolonged seizures
8. Low urinary output (<15 mL/h or <0.5 mL/kg/h) OR MEW or SEWS score >4
Personal history of active disease (at least one of)
• Advanced malignancy
• Chronic kidney disease
• Chronic heart failure
• Chronic obstructive pulmonary disease
• New cerebrovascular disease
• Myocardial infarction
• Moderate/severe liver disease
• Evidence of cognitive impairment (e.g. long-term mental disorders, dementia, behavioural alterations or disability from stroke)
• Previous hospitalisation in past year
• Repeat ICU admission at previous hospitalisation (associated with a fourfold increase in mortality)
• Evidence of frailty
• Unintentional or unexplained weight loss (10 lbs in past year)
• Self-reported exhaustion (felt that everything was an effort or felt could not get going at least 3 days in the past week)
• Weakness (low grip strength for writing or handling small objects, difficulty or inability to lift heavy objects >=4.5 kg)
• Slow walking speed (walks 4.5 m in >7 s)
• Inability for physical activity or new inability to stand
• Nursing home resident/in supported accommodation
• Proteinuria on a spot urine sample: Positive marker for chronic kidney disease & predictor of mortality: >30 mg albumin/g creatinine
• Abnormal ECG (atrial fibrillation, tachycardia, any other abnormal rhythm or ≥5 ectopics/min, changes to Q or ST waves

**Figure 1.**
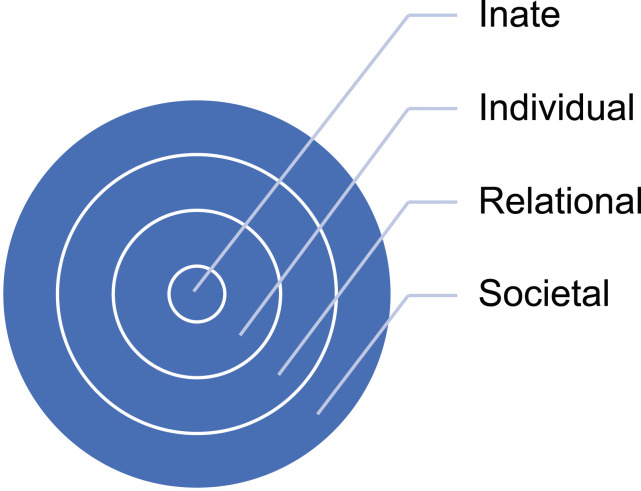
The ring theory of personhood^72^.

The second intervention proposed in response to a request from a patient for an assisted death, requires the adoption of a nuanced and holistic nursing response to the understanding of the dying patient and their interrelationships. The ring theory of personhood^72^ suggests that the dimension of a person is constituted by four interrelated dynamic areas (see Figure 1). The innate ring is innermost and anchored to the notion that all humans are deserving of personhood irrespective of their culture, creed, sexuality or clinical status. The individual ring, encasing the innate, stresses the importance of an individual’s unique values, beliefs, roles, preferences and ability to display consciousness. The third relational ring is defined by the individual’s close and personal ties. The outermost societal ring is characterised by societal as well as familial members, colleagues and acquaintances.

Both interventions are dependent on nursing staff embracing a holistic view of the dying patient through the promotion of open conversations within the context of valuing the interrelatedness of the patient to reframe patient autonomy whilst upholding patient choice. It is acknowledged that the administration of the crisTAL tool aligns with the NMC standards of proficiencies for registered nurses^23^ where there is a requirement to assess and review preferences, whilst recognising the role of nursing associates to review preferences and care priorities of the dying person and their family and carers 22.

## Conclusion

The situated nature of nursing practice is inextricably linked to perspectives of the morality of care informed by history^59^ and society^39^ that shape the landscape of practice contingent with the debate around assisted dying. The introduction of assisted dying would create a profound change to practice boundaries^35,67^ and practice learning pedagogy^73^ that may also threaten opportunities for researching end of life care using meaning-giving methods.^24^

The predominant ethical argument in favour of assisted dying driving calls for UK legislation appears to be patient choice and autonomy^74,75^ and is reflected in the conclusion of the House of Commons Health & Social Care committee inquiry report^1^ which stated dying people should be ‘…cared for with compassion and high-quality care and provided with as much agency and choice as possible’ 1 pg. 95.

This paper has highlighted the danger of members of the nursing profession coalescing around patient autonomy and individual choice as a singular predominant rationale for the acceptance of assisted dying; a clinical procedure that according to current evidence is deeply challenging to implement from a range of pharmacological, administrative, moral and emotional as well as ethical practice perspectives.
